# Maternal obesity and inadequate gestational weight gain disrupts placental dopaminergic signaling: impact on infant neurodevelopment

**DOI:** 10.3389/fendo.2026.1799454

**Published:** 2026-05-25

**Authors:** Nicole Soria-Reinoso, Arturo Flores-Pliego, Juan Mario Solis-Paredes, Arturo Alejandro Canul-Euan, Aurora Espejel-Nuñez, Teresa Hernández-Juárez, Sandra Martínez-Medina, Mariana Torres-Calapiz, Otilia Perichart-Perera, Carmen J. Zamora-Sánchez, Edgar Barrientos-Galeana, Héctor Borboa-Olivares, Guadalupe Estrada-Gutierrez, Ignacio Camacho-Arroyo

**Affiliations:** 1 Unidad de Investigación en Reproducción Humana, Instituto Nacional de Perinatología-Facultad de Química, Universidad Nacional Autónoma de México (UNAM), Mexico City, Mexico; 2 Department of Immunobiochemistry, Instituto Nacional de Perinatología, Mexico City, Mexico; 3 Department of Reproductive and Perinatal Health Research, Instituto Nacional de Perinatología, Mexico City, Mexico; 4 Centro de Estudios Avanzados sobre Violencia-Prevención (CEAVI-P), Instituto Nacional de Pediatría, Mexico City, Mexico; 5 Star Médica Hospital Infantil Privado, Mexico City, Mexico; 6 Department of Developmental Neurobiology, Instituto Nacional de Perinatología, Mexico City, Mexico; 7 Nutrition and Bioprogramming Coordination, Instituto Nacional de Perinatología, Mexico City, Mexico; 8 Community Interventions Research Branch, Instituto Nacional de Perinatología Isidro Espinosa de los Reyes, Mexico City, Mexico

**Keywords:** dopaminergic system, gestational weight gain, maternal obesity, neurodevelopment, neuroplacentology, placenta

## Abstract

**Introduction:**

The maternal-fetal axis plays a critical role in human development. Obesity during pregnancy may be associated with alterations in this axis, particularly the placental dopaminergic signaling, potentially affecting infant neurodevelopment.

**Methods:**

Participants (n=35) from the OBESO cohort (Mexico City) were classified by pregestational BMI as normal weight (n=20) or obese (n=15). Clinical and anthropometric variables of the mother–infant dyads were recorded. Neurodevelopment was evaluated in one-month-old infants using the Bayley-III Scales and classified as normal or altered (score ≤7 in any domain). Placental dopamine (DA) concentrations were quantified by ELISA and correlated with neurodevelopmental status. A subset of samples was analyzed by Western blot to explore the expression of key dopaminergic components: tyrosine hydroxylase (TH), catechol-O-methyltransferase (COMT), vesicular monoamine transporter 2 (VMAT2), organic cation transporter 3 (OCT3), dopamine transporter (DAT), and dopamine receptor D2 (DRD2).

**Results:**

Reduced placental DA concentrations, together with altered expression of key dopaminergic elements characterized by increased COMT and DAT, and decreased VMAT2, were observed in association with maternal obesity, inadequate gestational weight gain, and altered infant neurodevelopment.

**Conclusions:**

This study provides exploratory evidence that maternal obesity and inadequate GWG may be associated with early alterations in the intrauterine metabolic and neuroendocrine environment, including changes in the placental dopaminergic system. Differences in the expression of COMT, DAT, and VMAT2, together with lower placental DA concentrations, which may be linked to early neurodevelopmental variability in infants. Overall, the results suggest a potential relationship between maternal metabolic status and placental neuroendocrine pathways, warranting further investigation in larger and longitudinal studies.

## 1 Introduction

Obesity during pregnancy is a major public health concern since it is associated with multiple risk factors that compromise maternal-fetal health. Maternal obesity alters placental function, leading to increased lipid accumulation, elevated inflammatory markers, enhanced reactive oxygen species (ROS) production, and reduced antioxidant capacity (1–3) These alterations have been associated with changes in gene expression, mitochondrial metabolism, nutrient transport, and neurotransmitter production, including dopamine (DA), serotonin, and norepinephrine/epinephrine, which may influence maternal–fetal exchange and potentially impact the fetal brain development (3–5). Likewise, inadequate gestational weight gain (GWG), whether insufficient or excessive, has been reported to alter fetal brain neuromodulatory processes and increase the risk of neuro-developmental disorders, with effects varying according to the timing of exposure (6–8) Excessive GWG has been associated with elevated inflammatory markers, alterations in insulin and leptin resistance mechanisms, and disruptions in dopaminergic and serotonergic signaling, all of which have been linked to alterations in synaptic plasticity. In contrast, insufficient GWG, generally related to micronutrient deficiencies and protein malnutrition, compromises fetal neuronal proliferation during critical periods of early pregnancy (7, 9).

DA is a crucial neurotransmitter of the central and peripheral nervous systems. It is involved in various biological processes, and its signaling is essential for neurodevelopment, forebrain differentiation, and circuit formation. Placental DA is thought to play a role during the early stages of neurodevelopment, regulating the secretion of hormones such as human placental lactogen and gonadotropins, and contributing to neurogenesis, the maturation of axonal and dendritic projections, and the regulation of neuronal migration and differentiation, particularly within circuits related to reward, motivation, learning, and memory (4, 10–12).

DA is metabolized in the syncytiotrophoblast and can partially cross the placental barrier without being degraded, thereby reaching functionally relevant concentrations for fetal neurodevelopment (10, 13). DA is synthesized in the cytoplasm from the amino acid L-tyrosine (14). Tyrosine hydroxylase (TH), the rate-limiting enzyme in DA synthesis, has four isoforms, and its enzymatic activity can be inhibited by catecholamines or activated by kinases (15). The vesicular monoamine transporter 2 (VMAT2) packages DA into vesicles to facilitate its regulated release and to protect it from oxidative degradation in the cytoplasm (16, 17). In contrast, the DA transporter (DAT), which is Na^+^/Cl^-^-dependent, and the organic cation transporters (OCT1–3), recapture DA from the extracellular space for storage or metabolism (17, 18). The catechol-O-methyltransferase (COMT) degrades DA both inside and outside the cell through its soluble (S-COMT) and membrane-bound (MB-COMT) isoforms, with S-COMT being the predominant form in peripheral tissues (19, 20). Finally, DA activates multiple membrane receptors (DRD1–DRD5), triggering diverse transduction pathways and mediating various neuronal processes (11, 21).

Although several elements of the dopaminergic system have been identified in the human placenta (9, 21–24), their functional significance remains poorly defined, and the regulatory mechanisms governing their expression are still not fully understood. The placental dopaminergic system may be vulnerable to prenatal modifications induced by stressors such as maternal obesity or inadequate GWG, which could contribute to the increased risk of neurodevelopmental disorders, including autism spectrum disorder (ASD), attention-deficit/hyperactivity disorder (ADHD), epilepsy, and schizophrenia in children of women with obesity (23–25). However, the mechanistic links among maternal obesity, placental dopaminergic function, and long-term neurodevelopmental outcomes remain largely unknown. Accordingly, the objective of this study was to explore the relationship among maternal obesity, GWG, the placental dopaminergic system, and early infant neurodevelopment. We hypothesized that maternal obesity and altered GWG are associated with changes in placental dopaminergic signaling, which may be linked to early neurodevelopmental alterations.

## 2 Materials and methods

### 2.1 Participants

This study included pregnant women enrolled in the OBESO (Biochemical and Epigenetic Origins of Overweight and Obesity) perinatal cohort at the Instituto Nacional de Perinatología. This study was previously reviewed and approved by the Research Internal Review Board (INPer, Project Nos. 2021-1–11 and 2024-1-14), Mexico City, which has been previously described (26–28). Inclusion criteria were singleton pregnancy, absence of obstetric complications (gestational diabetes, preeclampsia, preterm birth), and absence of pregestational diabetes mellitus, renal or hepatic disease, as well as no treatment with glucocorticoids, anxiolytics, or antidepressants during gestation. We excluded women who were overweight (pregestational body mass index [pBMI] = 25-29.9 Kg/m^2^). Women were recruited at the Maternal Fetal Medicine Department during the first trimester of pregnancy (11-13.6 weeks). Participation was voluntary, and all women signed an informed consent. At the first visit, pregestational weight was self-reported by women, and current weight (digital scale: BMB-800, TANITA, Japan) and height (model 264, SECA, Hamburg, Germany) were also measured. pBMI was calculated, and women (n = 35) were classified as normal weight (BMI = 18.5-24.9; n = 20) or as having obesity (BMI ≥ 30; n = 15) according to World Health Organization criteria.

Clinical follow-ups included trimester biochemical assessments: glucose, triglycerides, cholesterol, high-density lipoprotein cholesterol (HDL-C), low-density lipoprotein cholesterol (LDL-C), and vitamin D were quantified in maternal plasma as previously described (26, 27, 29). GWG was calculated as the difference between maternal weight at the last prenatal visit and pregestational weight, and was categorized as insufficient, adequate, or excessive according to the Institute of Medicine’s 2009 guidelines (30).

Additionally, maternal age, gestational age at birth, mode of delivery, and the sex of the newborn were recorded, along with neonatal anthropometric measurements (31). Neonatal anthropometrics included: birth weight, length, ponderal index at birth, and head circumference, and were measured by trained dietitians using calibrated equipment during the first 48–72 h after delivery as previously described (28) Biochemical parameters were also assessed in fetal samples, including glucose, triglycerides, cholesterol, HDL-C, and LDL-C.

### 2.2 Infant neurodevelopmental assessment

Infant neurodevelopment was evaluated at one month of age using the Bayley Scales of Infant and Toddler Development, Third Edition (BSID-III). The one-month evaluation reported here corresponds to the baseline assessment of longitudinal neurodevelopmental follow-up schedule embedded in the OBESO cohort, which includes serial application of the BSID-III at 6, 12, 18, and 24 months (28). The one-month time point was purposefully selected to capture early postnatal signals proximally linked to *in utero* exposure, rather than to predict long-term neurodevelopmental outcomes, whose limited stability at this age is well recognized (32–34). Each assessment covered the cognitive, language (receptive and expressive), motor (fine and gross), and socio-emotional domains; the socio-emotional domain was assessed through a structured parent-completed questionnaire administered independently of the examiner. Scalar scores (mean = 10, SD = 3) were derived as specified in the test manual (32, 33). Neurodevelopment was classified *a priori* as altered when at least one domain obtained a scalar score ≤ 7—corresponding to performance > 1 SD below the normative mean—and as normal otherwise. The any-domain ≤ 7 cut-off has been proposed as a sensitivity-oriented screening threshold for early identification of infants at risk of developmental delay when the BSID-III is used for surveillance rather than diagnostic purposes (33, 34), and was selected a priori, before unblinding of biological data, to maximize sensitivity in light of (i) the floor effect and limited inter-domain differentiation of the BSID-III at one month and (ii) the small sample size, which would have rendered composite z-score approaches unstable. Raw scalar scores by domain are represented in **Supplementary Figure 1** (**Supplementary Material**) to allow inspection of the underlying distribution. Evaluations were performed by certified pediatric and developmental psychology specialists at INPer who were blinded to maternal pregestational BMI, gestational weight gain category, and to all placental laboratory results at the time of administration and scoring. Blinding was maintained until database lock, after which clinical and biological variables were merged for statistical analysis.

### 2.3 Placental tissue collection

Placental tissue samples (~2 cm³ from maternal and fetal sides) were immediately collected after delivery, transported under sterile, temperature-controlled conditions (4 °C), and stored at –80 °C until analysis. Placental tissue was rinsed with 1X PBS to remove erythrocytes, lysed, and homogenized at 4 °C using RIPA buffer (Cat. 89900, Thermo Fisher Scientific, Waltham, MA, USA) supplemented with Halt^®^ 1X protease inhibitor (Cat. 87786, Thermo Fisher Scientific, Waltham, MA, USA) until a uniform lysate was obtained. Samples were centrifuged at 14,000 rpm at 4 °C for 30 minutes. The supernatant was collected, and total protein concentration was quantified using the bicinchoninic acid (BCA) assay kit (Cat. 23227, Thermo Fisher Scientific, MA, USA) following the manufacturer’s instructions. For biochemical analyses, placental tissue obtained from the maternal and fetal sides was processed as a whole-tissue homogenate and analyzed as total placental lysate rather than as separately dissected regional fractions.

### 2.4 ELISA assays

Placental DA concentrations were quantified using a commercial DA ELISA Kit (Cat. KA1887, Abnova), following the manufacturer’s instructions. A total of 50 µL of placental extract was used for each reaction. All samples were acetylated and analyzed in duplicate. Absorbance was measured at 450 nm using a microplate reader (Synergy HT, Biotek Instruments, Winooski, VT, USA), and concentrations were calculated using a four-parameter logistic regression standard curve. The obtained values were normalized to the total protein content of the placental extract and expressed as ng/µg of protein. According to the manufacturer, the assay has an analytical range of 75–33,333 pg/mL for plasma, with a sensitivity of 49 pg/mL. The assay shows 100% specificity for dopamine, with reported cross-reactivity with other catecholamines, including norepinephrine (6.4%), 3-methoxytyramine (0.49%), and epinephrine (0.02%).

The assay was applied to human placental homogenates according to the manufacturer’s instructions; however, matrix-specific analytical validation in placental tissue was not independently established in our laboratory. Therefore, the results should be interpreted within the analytical limitations of a commercial ligand-binding assay applied to a complex tissue matrix. Due to inadequate placental tissue quality, DA quantification was not possible in two cases. Specifically, these samples did not meet the required criteria for protein integrity and homogenization, precluding reliable biochemical analysis. Consequently, these cases were excluded from DA-related analyses but remained included in all other clinical and perinatal assessments. Because all samples were analyzed in duplicate but formal intra- and inter-assay precision estimates were not independently established in this study, assay-related variability cannot be completely separated from biological variability.

### 2.5 Western blotting

Placental samples from the normal dyads (normal pBMI and adequate GWG whose infants showed normal neurodevelopment) and altered dyads (obesity and inadequate GWG whose infants exhibited altered neurodevelopment) were selected to evaluate the expression of dopaminergic system components by Western blot. Placental lysates from 9 cases were analyzed by Western blot. Samples were selected according to the predefined phenotypic classification of the study groups and based on adequate protein quality and quantity for biochemical analysis. A total of 5 µg of protein per lane was resolved by SDS-PAGE and transferred to PVDF membranes (Millipore, Billerica, MA, USA). Target proteins were detected using the following primary antibodies: rabbit monoclonal anti-TH (1:5000, Cat. Ab137869, Abcam, Cambridge, UK, England), rabbit polyclonal anti-COMT (1:5000, Cat. Ab129504, Abcam), rabbit monoclonal anti-DAT (1:800, Cat. Ab184451, Abcam), rabbit polyclonal anti-VMAT2 (1:1000, Cat. Nb11068123, Novus Biologicals), rabbit polyclonal anti-OCT3(1:1000, Cat. Ab183071, Abcam), rabbit polyclonal anti-DRD2 (1:800, Cat. Ab85367, Abcam), and rabbit polyclonal anti-GAPDH (1:10000, Cat. GTX100118, GeneTex, Irvine, CA, USA). Secondary antibodies included goat anti-rabbit IgG (1:2500, Cat. Ab6721, Abcam) and rabbit anti-mouse IgG (1:2500, Cat. Ab6728). Protein bands were visualized using SuperSignal™ West Femto chemiluminescent reagent (Thermo Scientific) and exposed to radiographic film. While this approach is widely used, it may limit the dynamic range and quantitative precision of signal detection and may affect the accuracy of densitometric quantification. To mitigate this, exposure conditions were optimized to avoid signal saturation. Densitometric quantification was performed using ImageJ software. Antibodies were used according to the manufacturers’ specifications and are described as detecting the corresponding target proteins; unless explicitly validated otherwise, they should not be interpreted as isoform-specific assays. GAPDH was used as a conventional loading control; however, its stability was not independently validated in this cohort, a limitation acknowledged. Densitometric analysis was performed using coded samples, and group allocation was revealed only after image quantification.

### 2.6 Statistical analysis

Clinical data were expressed as median [IQR], and experimental data as the mean ± standard error of the mean (SEM). Data distribution was assessed using the Shapiro–Wilk test, and homogeneity of variances was evaluated using Levene’s test to guide the selection of appropriate statistical methods. Comparisons of clinical variables and neurodevelopmental outcomes were performed using the Mann–Whitney U test for continuous variables, and Fisher’s test, for categorical variables. For categorical variables, odds ratios (OR) with 95% confidence intervals (CI) were estimated. DA concentrations were analyzed using the Kruskal-Wallis test, followed by Dunn’s *post hoc* test for multiple comparisons. Additionally, comparisons of placental DA concentrations between normal and altered mother–infant dyads were performed using Student’s t-test when normality assumptions were met. Western blot densitometry was evaluated using Student’s t-test or the Mann–Whitney U test, depending on data distribution. Effect sizes were calculated as Rosenthal’s r, using the standardized statistic (Z/√N) for non-parametric comparisons and derived from the t statistic for parametric analyses, and were interpreted as small (r ≈ 0.1), moderate (r ≈ 0.3), and large (r ≥ 0.5). Associations between placental DA, maternal parameters, and neurodevelopment were evaluated using Spearman correlation and simple regression analyses. For regression models, assumptions of linearity, independence of residuals, homoscedasticity, normality of residuals, and absence of influential outliers were assessed. Statistical significance was set at p<0.05. Effect sizes were interpreted as complementary measures to p-values, particularly given the exploratory nature of the study. All statistical analyses were performed using RStudio (version 2024.12.1 + 563; RStudio, PBC, Boston, MA, USA).

## 3 Results

### 3.1 Clinical characteristics of the study population

A total of 35 mother–infant dyads were included in the study. The mean age was 32.0 [29.5-34.5] years in the normal weight group and 32.0 [29.5–34.5] years in women with obesity, with no differences between two groups. Conversely, women with obesity had significantly higher pregestational weight and pBMI than women with normal weight (p < 0.001), as well as lower GWG (p = 0.014), as defined according to the Institute of Medicine’s 2009 guidelines. In addition, women with obesity showed higher third-trimester glucose concentrations (p = 0.036) and lower second-trimester LDL-C concentrations (p = 0.0118). No significant differences were observed in triglyceride, total cholesterol, and HDL-C concentrations across pBMI groups. Vitamin D concentrations were below the recommended range (< 30 ng/mL) in both groups. Complete values are presented in **Table 1** and in **Supplementary Table 1** (**Supplementary Material**).

**Table 1:** Clinical and biochemical characteristics of mothers according to pregestational BMI.

Variable	Normal weight (n=20)	Obesity (n=15)	P-value		Effect size
Age (years)	32.50 [29.00–35.00]	32.00 [29.50–34.50]	0.893		0.025
Pregestational weight (Kg)	55.00 [52.70–60.50]	82.00 [75.00–94.00]	<0.001	***	0.823
Stature (m)	1.58 [1.53–1.62]	1.58 [1.54–1.63]	0.688		0.070
pBMI (Kg/m²)	23.40 [22.30–24.15]	33.50 [30.40–36.35]	<0.001	***	0.845
GWG (Kg)	7.65 [6.20–12.38]	4.30 [1.45–8.50]	0.014	*	0.420
Insufficient	6.40 [5.60–6.60]	2.40 [-4.80–4.20]	0.006	**	0.691
Adequate	13.20 [12.10–13.40]	8.00 [6.75–8.50]	0.002	**	0.791
Excessive	16.75 [16.62–16.88]	11.20 [10.65–11.25]	0.15		0.775
Glucose T3 (mg/dL)	76.75 [68.75–83.67]	85.00 [79.50–89.90]	0.036	*	0.363
Triglycerides T3 (mg/dL)	197.00 [154.00–239.00]	207.50 [175.50–224.25]	1		0.003
LDL-C T2 (mg/dL)	123.85 [104.38–136.07]	103.40 [89.50–113.70]	0.0118	*	0.498

pBMI, Pregestational body mass index; GWG, Gestational weight gain. T2, T3, second and third trimesters, respectively; LDL, low-density lipoproteins. Continuous variables are expressed as median [interquartile range, IQR] and were compared using the Mann–Whitney U test. Effect size for continuous variables was calculated using Rosenthal’s r and was interpreted as follows: r < 0.10 = negligible, 0.10-0.29 = small, 0.30-0.49 = moderate, ≥0.50 = large. Statistical significance was set as: *(p<0.05), **(p<0.01), and *** (p< 0.001).

Regarding newborns, the mean gestational age at birth was 39.35 [38.35-40.10] weeks of gestation for newborns of normal-weight women, and 39.10 [38.05–39.55] weeks for newborns of women with obesity. Mean weight was 2.97 ± 0.1 Kg, and mean length 47.56 ± 0.5 cm. Clinical and metabolic parameters did not significantly differ by maternal weight status, and all measurements (weight, length, head circumference, and neonatal BMI) were within expected reference ranges for term infants. Similarly, fetal glucose and lipid profiles were comparable between groups, with all values remaining within normal reference ranges and no statistically significant differences detected. Detailed information is provided in **Supplementary Table 2** (**Supplementary Material**).

### 3.2 Neurodevelopmental alterations in infants from mothers with obesity and inadequate GWG

Neurodevelopment was assessed at one month of age. Overall, 28.5% of infants (n = 10) were classified as having normal neurodevelopment, whereas 71.4% (n = 25) were classified as having alterations in at least one neurodevelopmental domain. Domain-specific analysis showed that cognitive performance (28.6%; p = 0.011) and socio-emotional function (25.7%; p = 0.004) were the most significantly affected areas. Expressive language also showed a high percentage; however, it was not statistically significant (40%; p = 0.253). In contrast, receptive language and gross motor development showed alterations in only 2.9% of infants, and no deficits were observed in fine motor skills. See **Supplementary Figures 1, 2** (**Supplementary Material**).

**Figure 1:**
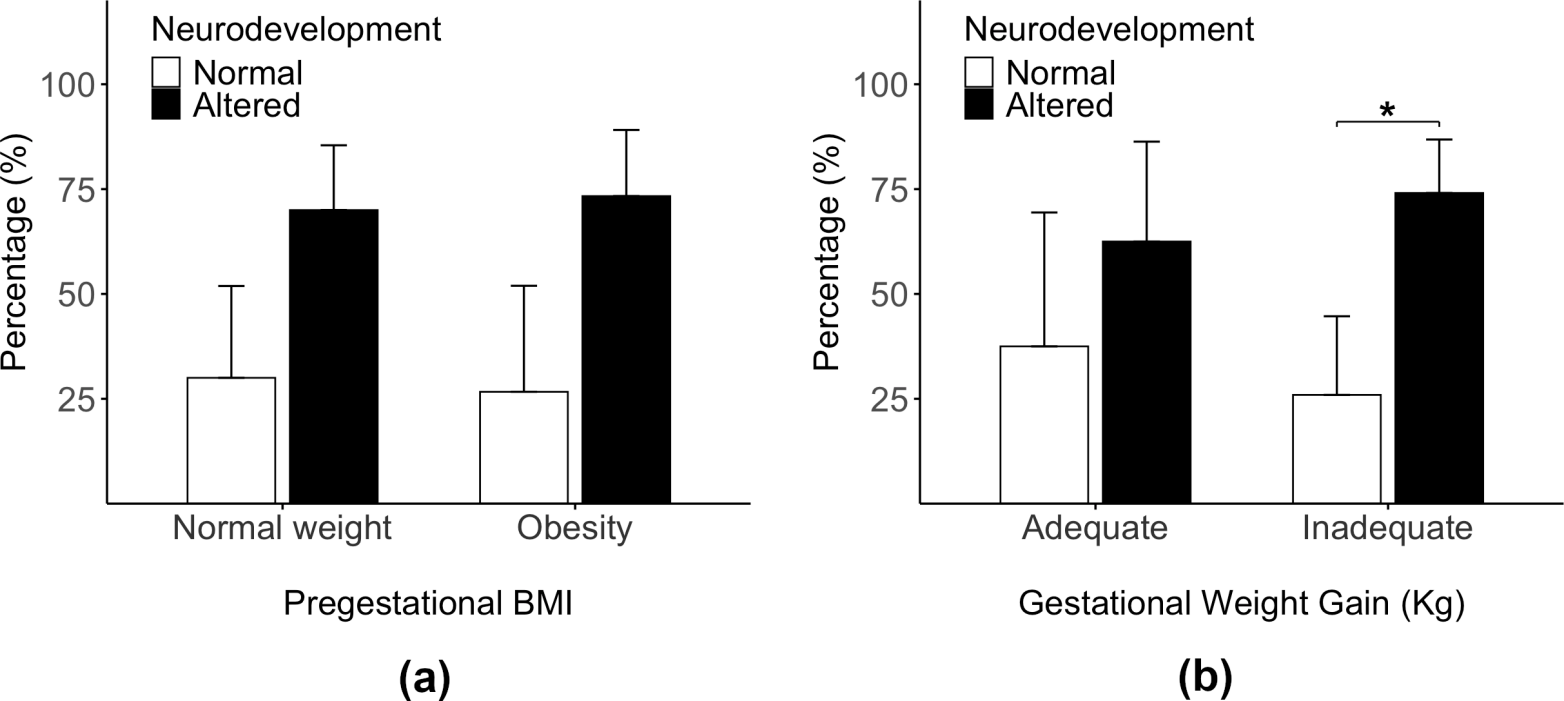
Neurodevelopmental outcomes according to pBMI and GWG. **(a)** Distribution of infant neurodevelopmental outcomes according to maternal pregestational BMI (n = 35): Normal weight (Normal = 6, Altered = 14) and Obesity (Normal = 4, Altered = 11). **(b)** Distribution of infant neurodevelopmental outcomes according to gestational weight gain (GWG): Adequate (Normal = 3, Altered = 5) and Inadequate, including insufficient and excessive GWG (Normal = 7, Altered = 20). Bars represent percentages, and error bars indicate 95% confidence intervals for proportions. Statistical differences in neurodevelopmental outcome distribution across maternal groups were evaluated using Fisher’s exact test (p < 0.05).

When the results were stratified by maternal pBMI, neurodevelopment was altered in infants from both normal-weight and pregnant women with obesity (**Figure 1a**; p = 1). Notably, in an independent analysis stratified by GWG, infants born to mothers with inadequate GWG exhibited a significantly higher proportion of altered neurodevelopment (**Figure 1b**; p = 0.022). These findings suggest that GWG may be more strongly associated with early neurodevelopmental variability than pregestational BMI.

### 3.3 Association of DA concentrations with maternal obesity, GWG, and infant neurodevelopment outcomes

**Figure 2:**
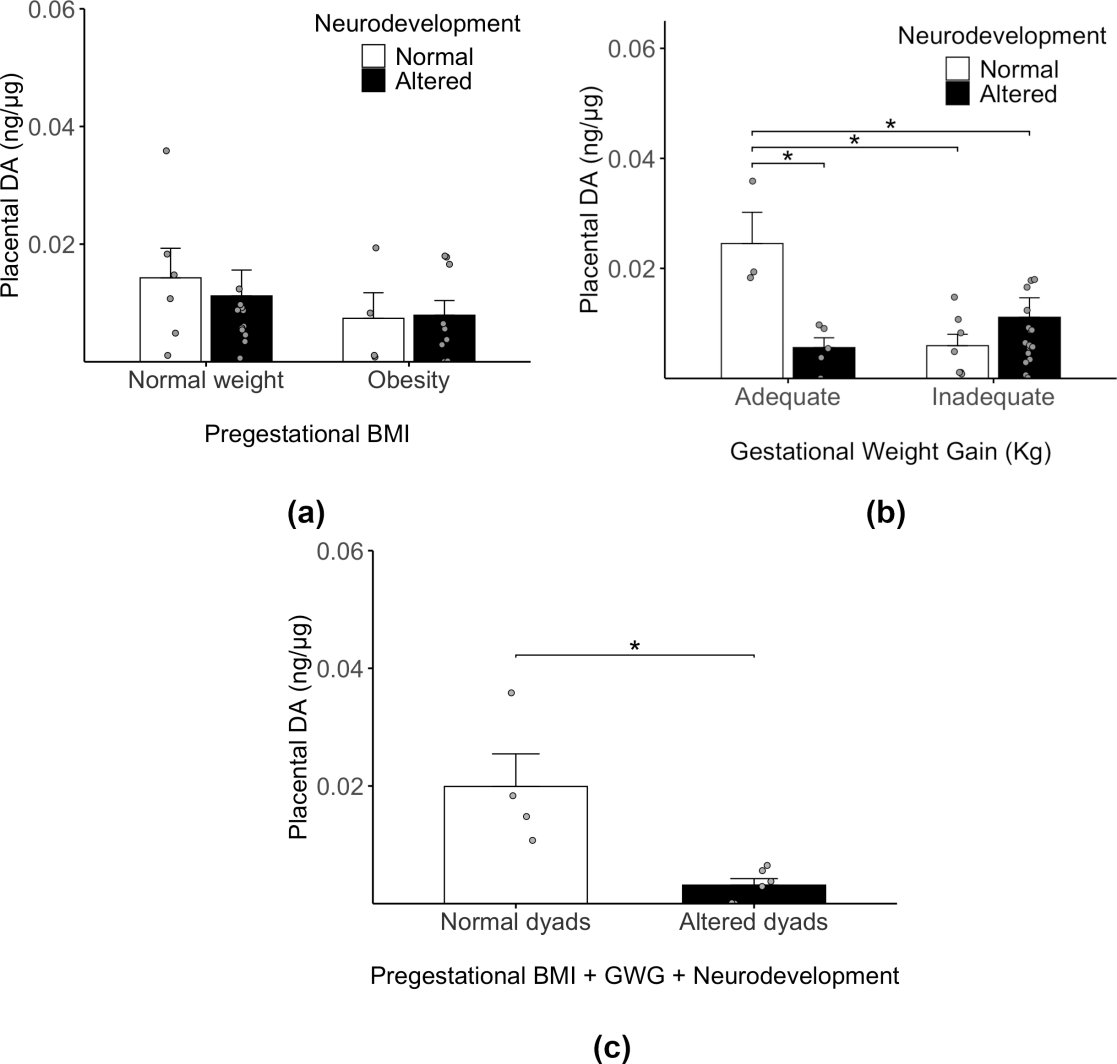
Placental dopamine (DA) concentrations in relation to maternal metabolic status and infant neurodevelopment. **(a)** Placental DA concentrations stratified by maternal pregestational BMI and infant neurodevelopmental status (n = 33; mean ± SEM). Groups based on maternal pregestational BMI: Normal weight (Normal neurodevelopment= 6, Altered neurodevelopment= 14); Obesity (Normal neurodevelopment = 4, Altered neurodevelopment = 9). Statistical differences were assessed using the Kruskal-Wallis test. **(b)** Placental dopamine (DA) concentrations according to GWG and infant neurodevelopmental status (n = 33; mean ± SEM). Groups: Adequate (Normal = 3, Altered = 5); Inadequate (Normal = 7, Altered = 18). Statistical differences were evaluated using the Kruskal-Wallis test, followed by Dunn’s *post hoc* test for multiple comparisons (*p ≤ 0.05). **(c)** Placental dopamine (DA) concentrations in normal versus altered mother–infant dyads. Normal dyads included mothers with normal pregestational BMI (Normal weight) and adequate gestational weight gain (GWG), whose infants showed normal neurodevelopment (n = 4). Altered dyads included mothers with obesity and inadequate GWG whose infants exhibited altered neurodevelopment (n = 5). Bars represent mean ± SEM. Statistical differences were assessed using Student’s t-test. (*p<0.05). Effect sizes and detailed pairwise comparisons are provided in **Supplementary Table 4** (**Supplementary Material**). Given the small sample size, these comparisons should be interpreted cautiously.

Placental DA concentrations ranged from 0.0000198 ng/µg to 0.0673 ng/µg. When stratified by pBMI (**Figure 2a**), both the normal-weight and obesity groups showed a high proportion of infants with altered neurodevelopment; however, no significant differences in placental DA concentrations were observed between the groups. When GWG was independently analyzed (**Figure 2b**), differences in placental DA concentrations were observed between women with adequate and inadequate GWG. This finding suggests an association between maternal GWG and placental DA concentrations; however, this analysis alone does not account for the observed neurodevelopmental variability in this cohort. Group differences, as well as *post hoc* comparisons that reached statistical significance, are presented in **Supplementary Table 4** (**Supplementary Material**).

Notably, when pBMI and GWG were simultaneously analysed (**Figure 2c**), a significant reduction in placental DA was observed in the altered dyads (obesity and inadequate GWG, whose infants exhibited altered neurodevelopment) compared with normal dyads (normal pBMI and adequate GWG whose infants showed normal neurodevelopment) (p = 0.05). These findings suggest that the combined effect of pregestational obesity and inadequate GWG was associated with lower placental DA concentrations, which may be linked to early neurodevelopmental variability.

**Figure 3:**
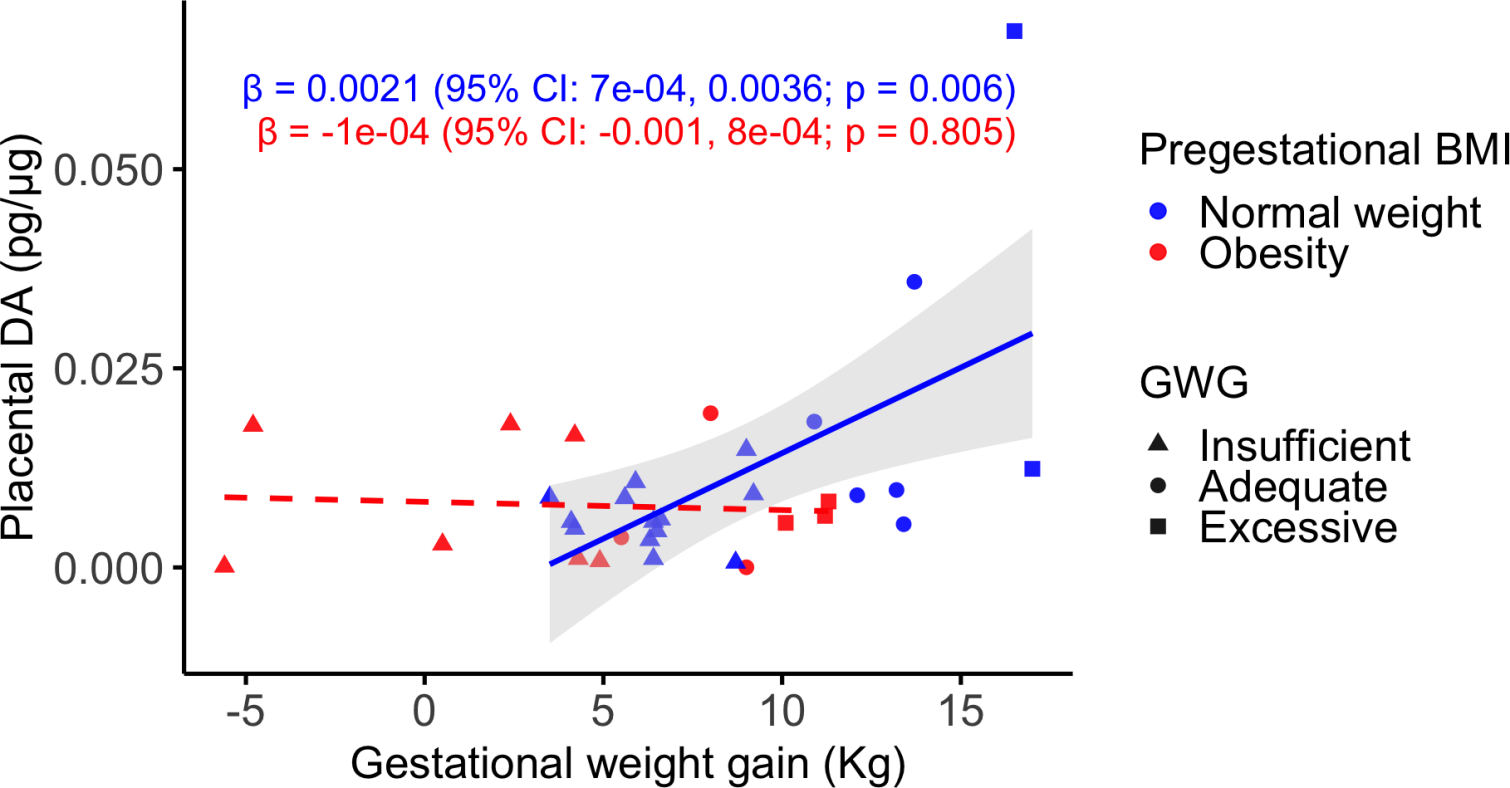
Association between GWG and placental DA concentrations according to maternal pBMI. A significant positive association between GWG and placental DA concentrations was observed only in the normal-weight group, whereas no significant association was detected in women with obesity.

Given the potential influence of GWG on neurodevelopmental outcomes, we evaluated the relationship between GWG and placental DA concentrations. Linear regression analyses revealed that, among normal-weight women, placental DA concentrations were positively associated with GWG, independent of neurodevelopmental status (**Figure 3**). In contrast, this association was not observed in pregnant women with obesity, suggesting that excess maternal adiposity may be associated with alterations in the physiological relationship between gestational growth and placental dopaminergic signalling.

### 3.4 Expression of dopaminergic system components

**Figure 4:**
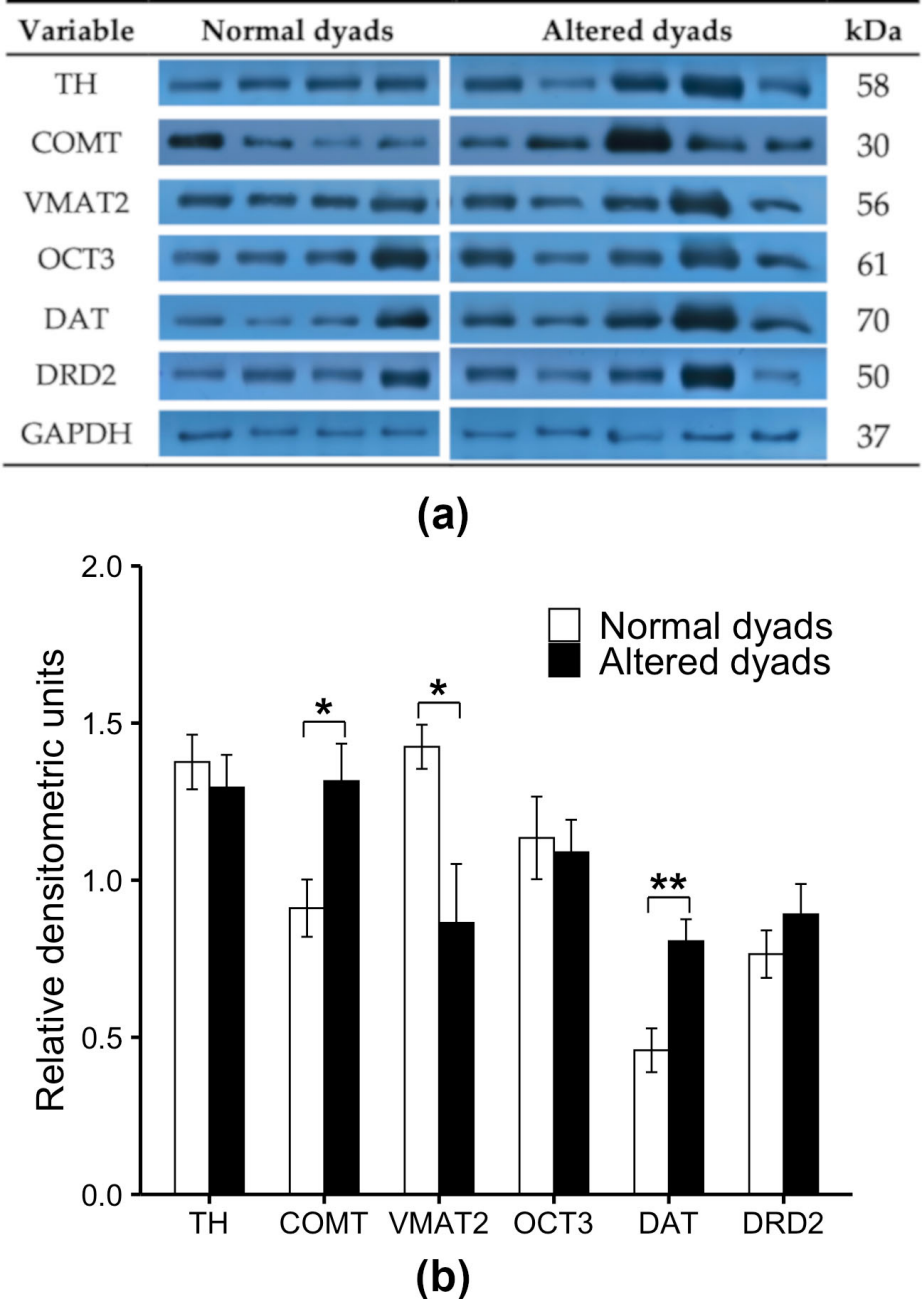
Expression of placental dopaminergic system components in normal and altered dyads. **(a)** Representative Western blot images of placental lysates from normal and altered dyads (n=9). Proteins evaluated include tyrosine hydroxylase (TH), catechol-O-methyltransferase (COMT), vesicular monoamine transporter 2 (VMAT2), organic cation transporter 3 (OCT3), dopamine transporter (DAT), and dopamine receptor D2 (DRD2). Bands were visualized by chemiluminescence and captured on radiographic film. **(b)** Densitometric quantification of TH, COMT, VMAT2, OCT3, DAT, and DRD2 expression, normalized to GAPDH. Statistical comparisons were performed using either Student’s t-test or the Mann–Whitney U test, depending on data distribution and variance assumptions. Data are presented as mean ± SEM. *(p < 0.05); **(p < 0.01). Effect sizes and detailed pairwise comparisons are provided in **Supplementary Table 5** (**Supplementary Material**).

The analysis of components of the placental dopaminergic system revealed selective alterations associated with the maternal–infant condition. Altered dyads showed a significant reduction in VMAT2 expression compared with normal dyads (p < 0.05). However, further assays are needed to demonstrate a potential reduction in vesicular DA storage capacity. In contrast, COMT and DAT content were significantly higher in altered dyads (p < 0.01), which may reflect alterations in pathways related to DA metabolism and reuptake. No significant differences were found in TH, OCT3, or DRD2 expression between groups (**Figure 4**).

## 4 Discussion

During the first 1000 days of life, a highly critical developmental period encompassing conception through the first two years of age, the brain undergoes rapid structural and functional changes, making it particularly vulnerable to adverse nutritional and environmental factors. In this context, metabolic alterations associated with maternal obesity and inadequate GWG may be linked to changes in neurogenesis and synaptogenesis through mechanisms such as inflammation, oxidative stress, lipotoxicity, and altered placental transfer of nutrients and neurotransmitters (25, 28). In this study, we highlight the potential relevance of exploring the role of the placental dopaminergic system as a neurodevelopmental regulator in the context of obesity and inadequate GWG in a hypothesis-driven way.

As long as obesity prevalence continues rising worldwide, especially in women of reproductive age, the most efforts must be made in understanding the potential risk of this condition to fetal programming, particularly to neurodevelopment. Here, we focused on evaluating neurodevelopmental state at a very early postnatal stage, as it is less influenced by covariates such as lactation status, ambient enrichment, and maternal attachment. However, it is important to distinguish this early neurodevelopmental variability from alterations with predictive clinical relevance. Given the early timing of assessment (1 month), the observed differences may reflect transient developmental variability rather than persistent neurodevelopmental impairment, and their long-term significance remains to be determined. Our findings suggest that maternal obesity and inadequate GWG may be associated with alterations in the intrauterine metabolic environment (**Table 1**; **Supplementary Table 1**) and with early neurodevelopmental variability in infants (6, 25, 35, 36). In this context, placental DA becomes particularly relevant, as it regulates maternal–fetal exchange and modulates essential cellular processes required for fetal neural maturation, including neural progenitor proliferation and differentiation, neuronal migration, and early synaptic circuit formation (4, 23, 37, 38). The observed placental protein expression patterns could be early indicators of an altered state of the placental dopaminergic system. However, the underlying mechanisms require further exploration to determine whether the observed changes in protein levels correlate with a disrupted functional state of the placental dopaminergic system.

Placental DA concentrations observed in this study cannot be directly compared with those reported in isolated human syncytiotrophoblast vesicles (10), as these correspond to subcellular fractions rather than whole-tissue extracts. Differences in sample type and analytical approach may partly explain these discrepancies. In this context, the placental DA concentrations observed in this study (0.0104 ng/µg) fall within the range reported for peripheral tissues (39), supporting the biological plausibility of the measured values. To our knowledge, this is the first study to report dopamine concentrations in whole human placental tissue normalized to total protein content. These findings provide initial evidence supporting the presence of a dopaminergic system at the placental level and its potential relevance in maternal metabolic conditions, although they should be interpreted within an exploratory framework.

Previous studies have shown that maternal obesity has been associated with alterations in dopaminergic signalling in offspring, primarily through placental inflammation and oxidative stress (23, 24, 40, 41). In our study, we observed lower placental DA concentrations in altered dyads (obesity and inadequate GWG whose infants exhibited altered neurodevelopment) compared to normal dyads (**Figure 2c**). This observation is consistent with animal models in which maternal high-fat diets have been associated with changes in dopaminergic neurotransmission, including reduced DA release and reuptake, thereby decreasing extracellular DA concentrations in regions such as the nucleus accumbens (42, 43). In nonhuman primate offspring exposed to a maternal high-fat diet and obesity, these alterations have been associated with differences in dopaminergic projections to the prefrontal cortex and with behavioral outcomes (44), although direct extrapolation to human placental function should be made cautiously.

In line with this, we found that inadequate GWG was associated with adverse perinatal outcomes and a higher likelihood of infant neurodevelopmental alterations (**Figure 1b**) (25, 45). Notably, significant differences in placental DA concentrations were observed between the Adequate GWG - Normal neurodevelopment, and Inadequate GWG - Altered neurodevelopment groups (**Figure 2b**), suggesting a potential relationship between GWG and placental DA availability. Moreover, a positive correlation between GWG and placental DA concentrations was observed in women with normal weight (**Figure 3**). However, these differences were less pronounced than those observed when GWG alterations occurred in conjunction with maternal obesity, indicating that GWG alone may not fully explain the dopaminergic and neurodevelopmental alterations, which need to be explored further. Consistently, studies in murine models have shown that maternal high-fat diets have been associated with alterations in the offspring’s dopaminergic system, with late-gestation exposure leading to reduced striatal DA concentrations and distinct neurochemical adaptations compared with early-gestation exposure. These experimental data are consistent with the possibility that maternal nutritional status during pregnancy influences the development of fetal dopaminergic pathways, aligning with our observation that altered GWG may contribute to early neurodevelopmental vulnerability in human infants (46).

In this context, the observed imbalance in the expression of components of the placental dopaminergic system is consistent with altered DA homeostasis within the feto-placental unit, which has been associated, in experimental models, with disturbances in essential processes such as neurogenesis, angiogenesis, and synaptogenesis during critical periods of fetal brain development (4, 47, 48). Whether the protein expression patterns described here translate into functional disruption of these processes in human infants remains to be established.

**Figure 5:**
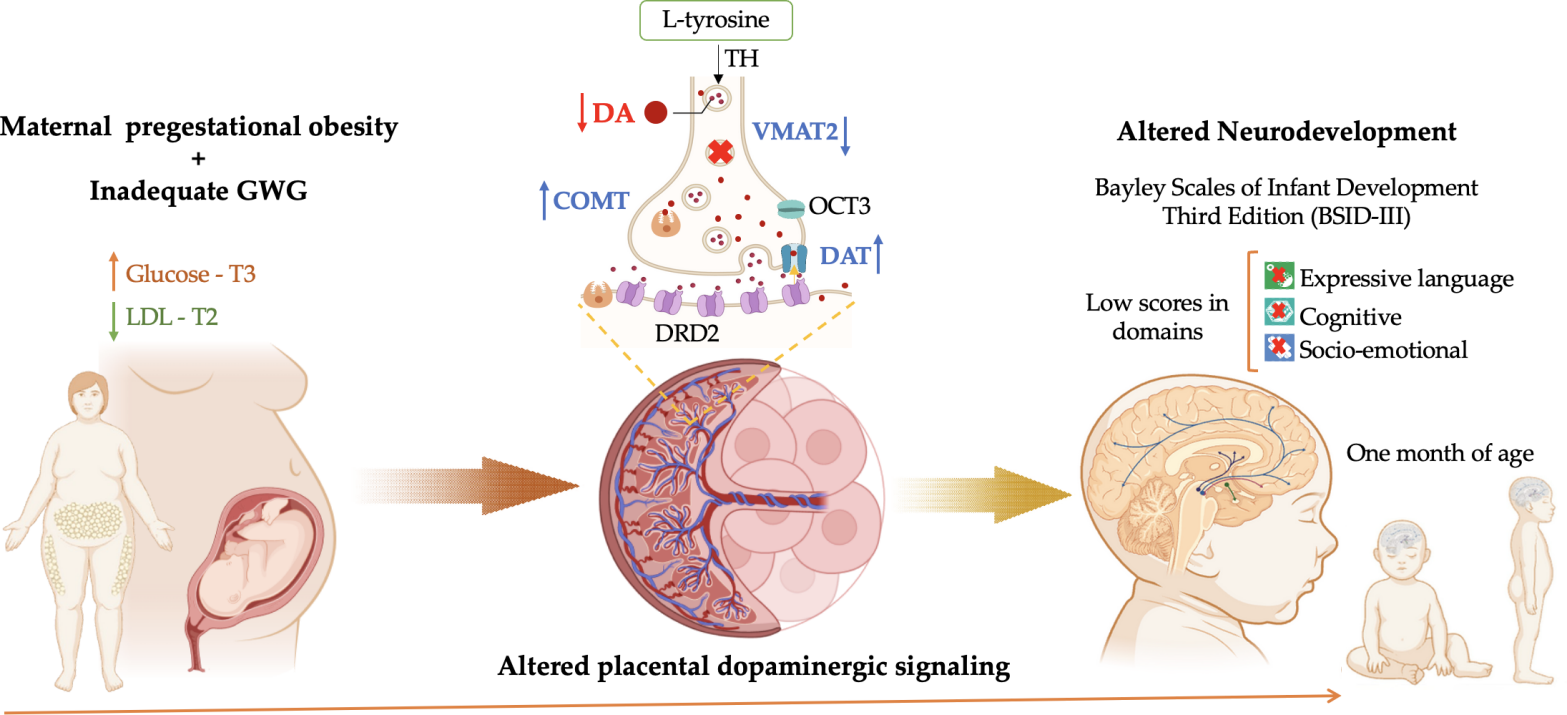
Proposed model of placental dopaminergic alterations associated with maternal obesity and inadequate GWG. Maternal obesity and inadequate GWG may be associated with alterations in placental dopamine (DA) homeostasis through coordinated changes in key dopaminergic regulators. Increased catechol-O-methyltransferase (COMT) expression may contribute to enhanced DA degradation, while reduced vesicular monoamine transporter 2 (VMAT2) expression may be associated with impaired DA vesicular storage. Increased dopamine transporter (DAT) expression may reflect changes in DA reuptake and clearance. Collectively, these alterations may influence placental DA availability at the maternal–fetal interface and could be linked to processes involved in fetal brain development, including neuronal maturation and circuit formation. This model represents a hypothetical and exploratory framework derived from associative findings that need to be addressed. Created with BioRender.com.

In our study, we observed increased COMT and DAT, along with reduced VMAT2 expression, in the altered dyads (**Figure 4**). These findings are supported by studies reporting increased phosphorylated COMT expression in the placenta and fetal liver of animals exposed to maternal obesity (49), as well as overexpression of genes involved in DA availability (Th and Slc6a3/Dat1) in the brains of offspring fed with a high-fat diet (50). Additionally, maternal obesity has been shown to alter the expression of nutrient and monoamine transporters in the placenta due to a pro-inflammatory and lipotoxic microenvironment (51–53), which may explain reduced VMAT2 expression and, consequently, may imply an inefficient DA storage. DAT expression in the human placenta has been reported to be low and with limited functional relevance (10); however, our findings suggest that its overexpression may reflect a potential compensatory mechanism (54), more analysis is needed to demonstrate a decreased vesicular storage of DA or increased DA catabolism (**Figure 5**) (55).

Together, these findings are compatible with a coordinated alteration of key elements of the placental dopaminergic system—specifically increased COMT and DAT, decreased VMAT2, and reduced placental DA concentrations—in mother–infant dyads exposed to pregestational obesity and inadequate GWG. Within the limits of a cross-sectional, expression-level analysis, this pattern represents a potential marker of an altered placental dopaminergic state rather than a validated functional mechanism, and the inference of impaired fetal DA exposure should be regarded as speculative pending direct functional and longitudinal evidence (**Figure 5**).

Within the conceptual framework of fetal programming, our findings are consistent with the hypothesis that maternal obesity and inadequate GWG may be associated with early disturbances in placental dopaminergic signaling. Whether these disturbances exert long-lasting effects on infant neurodevelopment is unknown and cannot be inferred from the present cross-sectional, one-month assessment. This question is particularly relevant because, during early fetal brain development, the placenta is the primary source of catecholamines, and toward the end of gestation it acquires a modulatory role on fetal exposure to circulating DA concentrations (47); accordingly, dysregulation of this system may be associated with altered availability of DA at key moments of neuronal maturation, a hypothesis that requires longitudinal confirmation.

These findings may position the placental dopaminergic system as a candidate early biological signal of neurodevelopmental vulnerability in infants exposed to maternal obesity and inadequate GWG. Its potential role as an early biomarker or therapeutic target is, however, premature at this stage and should be regarded as a future research direction requiring validation in larger, longitudinal cohorts with functional assays and prospective neurodevelopmental follow-up. From a clinical perspective, the results reinforce the broader rationale for strengthening prenatal metabolic monitoring and optimizing gestational weight trajectories, while the incorporation of placental neuroendocrine markers in risk-stratification strategies awaits formal validation.

To our knowledge, this is one of the first studies in a Latin American population to explore the relationship between maternal metabolic status, placental dopaminergic profiles, and early neurodevelopment. Conducted within a population with a high prevalence of metabolic risk during pregnancy, these findings provide regionally relevant data and support the need for further research in diverse populations to better understand the role of placental neuroendocrine pathways in early development.

This study has several limitations that should be considered when interpreting the findings. First, the sample size is modest, which limits statistical power, the stability of subgroup estimates, and the generalizability of the results precludes adjustment for additional covariates. Second, neurodevelopment was assessed at one month of age, a developmental window in which BSID-III scores have limited predictive validity for long-term cognitive, language, or motor outcomes (32–34); the present findings should therefore be interpreted as early neurobiological signals rather than as predictive markers of later neurodevelopmental impairment. The classification of infants as altered using an any-domain scalar score ≤ 7, although chosen *a priori* for sensitivity, collapses domains with distinct biological underpinnings and increases the risk of false-positive classification; consequently, our findings should not be interpreted as domain-specific deficits, but rather as exploratory signals warranting longitudinal confirmation. The persistence and clinical significance of these early signals are unknown and are being prospectively assessed within longitudinal follow-up of the OBESO cohort, whose neurodevelopmental trajectories during the first year of life have been previously reported (28). Third, placental DA concentrations were measured in whole-tissue lysates collected at delivery, which may not fully capture compartment-specific dynamics, regional gradients within the placental disc, or earlier gestational fluctuations in dopaminergic signaling. In addition, because placental tissue from the maternal and fetal sides was analyzed as a whole-tissue homogenate rather than as separately dissected regions, our findings should be interpreted as integrated tissue-level measurements. Given the known regional heterogeneity of the human placenta, this represents a limitation that may affect biological comparability and reproducibility across studies. Fourth, our Western blot data inform on steady-state protein abundance and not on transporter activity, DA uptake/release kinetics, or enzymatic turnover; direct functional assays were beyond the scope of this exploratory study and are proposed as future work. Despite these limitations, the study provides novel human evidence supporting an associative link between maternal obesity, inadequate gestational weight gain, and placental dopaminergic dysregulation impacting neurodevelopment, and constitutes a hypothesis-generating framework for larger longitudinal studies. Consistent with this, previous studies have reported high percentages of altered development in specific domains assessed using the Bayley Scales at different ages (34, 56). Another important consideration for future studies on the dopaminergic system in the placenta is that fetal sex may also be a key variable to consider, which we do not explore due to the sample size of the study. However, some authors have shown that placental transcriptomics, structure, and function are influenced by the fetal sex (57, 58).

Finally, larger longitudinal studies are needed to confirm whether placental dopaminergic alterations predict later neurodevelopmental outcomes. Integrating molecular profiling of placental neurotransmitter pathways with maternal metabolic markers may help identify mechanistic targets and guide the development of early interventions to mitigate neurodevelopmental risk.

## 5 Conclusions

In summary, this study provides exploratory evidence that maternal obesity and inadequate GWG may be associated with early alterations in the intrauterine metabolic and neuroendocrine environment, including changes in the placental dopaminergic system and alterations in neurodevelopment. Differences in the expression of COMT, DAT, and VMAT2, together with lower placental DA concentrations, may reflect alterations in pathways related to DA homeostasis at the maternal–fetal interface. These alterations may be linked to early neurodevelopmental outcomes in infants; however, causal relationships cannot be established due to the cross-sectional design and the early timing of neurodevelopmental assessment.

Overall, these findings are consistent with the DOHaD paradigm, suggesting that maternal metabolic and neuroendocrine conditions may influence early neurodevelopment through placental pathways. The placental dopaminergic system may represent a candidate biological signal that warrants further investigation, although its potential role as a biomarker or therapeutic target remains to be validated. Further research in larger, longitudinal cohorts with functional approaches is required to determine the clinical relevance of these findings.

## Data Availability

The raw data supporting the conclusions of this article will be made available by the authors, without undue reservation.
