# Supplementary material for: Maternal obesity and inadequate gestational weight gain disrupts placental dopaminergic signaling: impact on infant neurodevelopment
Source: Front Endocrinol (Lausanne). 2026 May 25;17:1799454. doi: 10.3389/fendo.2026.1799454 (PMC13243056; doi:10.3389/fendo.2026.1799454)
Supplement: Supplementary file 1 [file DataSheet1.docx]

**Table S1.** Biochemical characteristics of pregnant women according to pregestational BMI.

| Variable | Normal weight (n=20) | Obesity (n=15) | p_value | | Effect size |
| --- | --- | --- | --- | --- | --- |
| *Glucose (mg/dL)* |  |  |  |  |  |
| *T1* | 76.60 [68.22–84.03] | 84.20 [73.65–89.20] | 0.152 |  | 0.245 |
| *T2* | 72.75 [64.33–81.83] | 77.00 [72.70–83.20] | 0.386 |  | 0.149 |
| *T3* | **76.75 [68.75–83.67]** | **85.00 [79.50–89.90]** | **0.036** | ***** | **0.363** |
| *Triglycerides (mg/dL)* | |  |  |  |  |
| *T1* | 113.50 [89.75–160.25] | 125.00 [115.00–150.00] | 0.537 |  | 0.107 |
| *T2* | 152.50 [123.25–179.75] | 175.00 [151.50–180.50] | 0.25 |  | 0.197 |
| *T3* | 197.00 [154.00–239.00] | 207.50 [175.50–224.25] | 1 |  | 0.003 |
| *Cholesterol (mg/dL)* | |  |  |  |  |
| *T1* | 179.00 [164.00–190.75] | 178.00 [167.50–198.00] | 0.726 |  | 0.062 |
| *T2* | 220.50 [194.00–249.00] | 200.00 [186.00–229.00] | 0.156 |  | 0.242 |
| *T3* | 234.00 [206.00–288.00] | 237.00 [193.25–276.25] | 0.766 |  | 0.054 |
| *HDL-C (mg/dL)* |  |  |  |  |  |
| *T1* | 56.15 [52.50–64.10] | 59.40 [51.80–70.30] | 0.79 |  | 0.048 |
| *T2* | 61.30 [58.02–70.93] | 62.50 [50.35–73.25] | 0.677 |  | 0.073 |
| *T3* | 60.20 [56.85–70.42] | 62.55 [50.30–76.22] | 0.916 |  | 0.021 |
| *LDL-C (mg/dL)* |  |  |  |  |  |
| *T1* | 79.60 [69.95–96.08] | 88.40 [72.80–104.50] | 0.405 |  | 0.144 |
| *T2* | **123.85 [104.38–136.07]** | **103.40 [89.50–113.70]** | **0.012** | ***** | **0.498** |
| *T3* | 130.35 [102.98–142.85] | 125.90 [103.40–145.30] | 0.93 |  | 0.018 |
| *Vitamin D (ng/mL)* | |  |  |  |  |
| *T1* | 22.50 [20.60–24.85] | 17.70 [15.60–23.20] | 0.111 |  | 0.280 |
| *T2* | 27.65 [20.23–32.02] | 23.30 [20.05–31.00] | 0.377 |  | 0.152 |
| *T3* | 27.40 [21.18–31.75] | 22.50 [18.10–37.35] | 0.612 |  | 0.090 |

**Note**: pBMI: Pregestational body mass index; GWG: Gestational weight gain. T1, T2, T3: first, second, and third trimester, respectively; HDL: high-density lipoproteins; LDL: low-density lipoproteins. Continuous variables are expressed as median [interquartile range, IQR] and were compared using the Mann–Whitney U test. Effect size for continuous variables was calculated using Rosenthal’s r and was interpreted as follows: r < 0.10 = negligible, 0.10-0.29 = small, 0.30-0.49 = moderate, ≥0.50 = large. Statistical significance was set as: *****(p<0.05), ******(p<0.01), and ******* (p< 0.001).

**Table S2.** Clinical and biochemical characteristics of infants according to maternal pregestational BMI.

| **Variable** | **Normal weight (n=20)** | **Obesity (n=15)** | **p_value** | | **Effect size** |
| --- | --- | --- | --- | --- | --- |
| *Mode of delivery – n (%)* |  |  | |  |  |
| *1. Cesarean section* | 16 (80%) | 8 (53.3%) | | *****0.144 | OR: 3.37  IC95: 0.63-20.87 |
| *2. Vaginal* | 4 (20%) | 7 (46.7%) | |  |  |
| *Sex – n (%)* |  |  | |  |  |
| *1. Male* | 11 (55%) | 10 (66.7%) | | *****0.727 | OR: 0.62  IC95: 0.12-2.97 |
| *2. Female* | 9 (45%) | 5 (33.3%) | |  |  |
| *GA at birth (weeks)* | 39.35 [38.35–40.10] | 39.10 [38.05–39.55] | | 0.463 | 0.124 |
| *Birth weight (g)* | 3.05 [2.85–3.12] | 2.80 [2.63–3.17] | | 0.484 | 0.118 |
| *Birth height (cm)* | 47.88 [47.11–48.71] | 46.65 [45.95–48.98] | | 0.582 | 0.093 |
| *Ponderal index at birth (Kg/m³)* | 13.00 [12.33–13.83] | 12.60 [12.15–13.95] | | 0.726 | 0.059 |
| *HC at birth (cm)* | 34.10 [33.15–34.50] | 32.65 [32.42–34.58] | | 0.353 | 0.159 |
| *Fetal glucose (mg/dL)* | 51.10 [41.10–70.90] | 40.30 [31.75–53.85] | | 0.18 | 0.253 |
| *Fetal triglycerides (mg/dL)* | 35.00 [28.00–45.00] | 21.00 [16.00–37.50] | | 0.158 | 0.267 |
| *Fetal cholesterol (mg/dL)* | 58.00 [50.00–76.00] | 54.00 [51.00–63.50] | | 0.588 | 0.102 |
| *Fetal HDL-C (mg/dL)* | 27.70 [22.80–34.38] | 25.30 [21.60–36.35] | | 0.805 | 0.049 |
| *Fetal LDL-C (mg/dL)* | 25.10 [24.50–31.40] | 24.30 [21.45–26.40] | | 0.224 | 0.248 |

**Note:** GA: Gestational age; HC: Head circumference; HDL-C: High-density lipoprotein cholesterol; LDL-C: Low-density lipoprotein cholesterol. Continuous variables are expressed as median [interquartile range] and compared using the Mann–Whitney U test. Effect size for continuous variables was calculated using Rosenthal’s r. Effect size was interpreted as follows: r < 0.10 = negligible, 0.10–0.29 = small, 0.30–0.49 = moderate, ≥ 0.50 = large. Categorical variables and proportions were compared using Fisher’s test and are presented as odds ratios (OR) with 95% confidence intervals (95% CI).

**Table S3.** Concordance between pregestational BMI classification and first-trimester BMI classification.

| **Pregestational BMI** | **Normal weight T1** | **Obesity T1** | **Overweight T1** | **Total** |
| --- | --- | --- | --- | --- |
| Normal weight | 16 | 0 | 4 | 20 |
| Obesity | 0 | 14 | 1 | 15 |
| Total | 16 | 14 | 5 | 35 |

**Note:** Agreement between pregestational BMI classification (based on self-reported weight) and first-trimester BMI classification (based on measured weight) was 85.7%, with a Cohen’s kappa coefficient of 0.75, indicating substantial concordance. A total of 5 participants (14.23%) were reclassified between categories. Importantly, all reclassifications occurred toward the intermediate category (overweight), with no transitions observed between normal weight and obesity. Due to the relatively small sample size and sparse data in some cells, confidence intervals for Cohen’s kappa should be interpreted with caution.


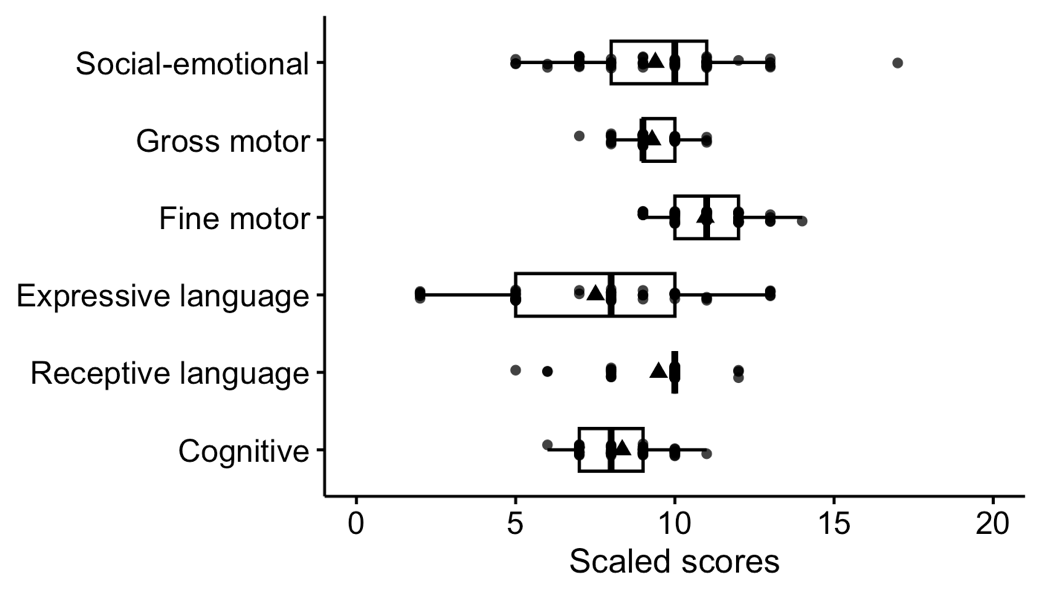


**Figure S1. Distribution of Bayley-III scaled scores across neurodevelopmental domains.**
Box-and-whisker plots showing the distribution of individual scaled scores for cognitive, receptive language, expressive language, fine motor, gross motor, and social-emotional domains assessed using the Bayley Scales of Infant and Toddler Development, Third Edition (Bayley-III). Boxes represent the interquartile range (IQR) with the median indicated by a central line, whiskers denote minimum and maximum values, and all individual observations are displayed. Triangles indicate mean values. Scaled scores are standardized (mean = 10, SD = 3).


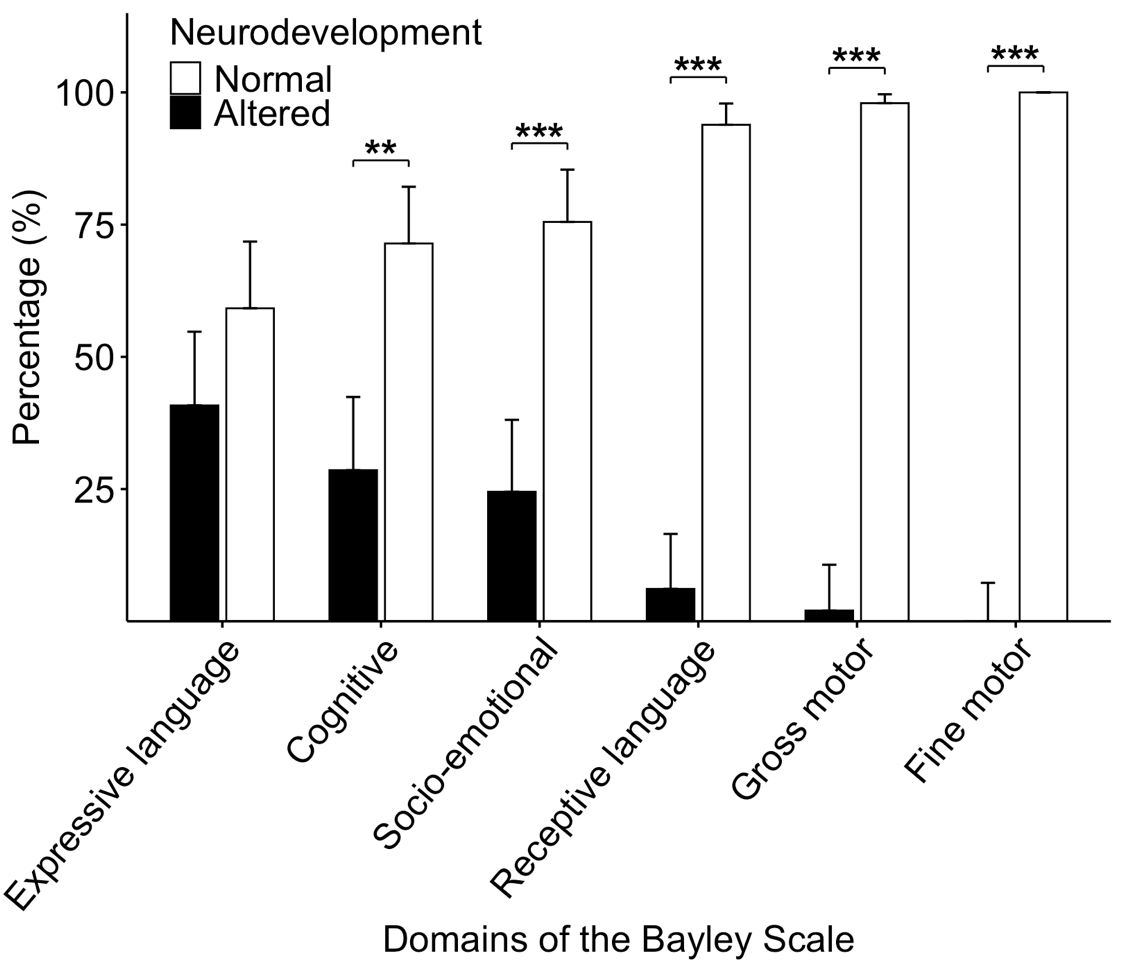


**Figure S2. Neurodevelopmental classification across Bayley-III domains (n = 35).**

Percentage of infants classified as having normal or altered performance in each Bayley-III domain, including expressive language, cognitive, socio-emotional, receptive language, gross motor, and fine motor domains. Error bars represent 95% confidence intervals for binomial proportions (Wilson method), displayed only above each bar. Although most infants exhibited normal performance within individual domains, overall neurodevelopmental status was defined at the individual level: infants were classified as having altered neurodevelopment if at least one domain showed an altered score. Under this criterion, a higher proportion of infants were categorized as having altered neurodevelopment (71.4%). Statistical differences between normal and altered outcomes within each domain were evaluated using binomial tests (p < 0.05; **p < 0.01; ***p < 0.001).

**Table S4.** Pairwise comparisons of placental DA concentrations according to pregestational BMI, GWG, and neurodevelopmental status, including effect size estimates.

| **Group 1 (n)** | **Group 2 (n)** | **p_value** | **Effect size** |
| --- | --- | --- | --- |
| *Pregestational BMI + Neurodevelopment* | | | |
| Normal weight_Normal (6) | Normal weight_Altered (14) | 0.303 | 0.240 |
| Obesity_Normal (4) | Obesity_Altered (9) | 0.939 | 0.043 |
| *GWG + Neurodevelopment* | | | |
| Adequate _Altered (5) | Inadequate _Altered (18) | 0.508 | 0.833 |
| Adequate _Normal (3) | Inadequate _Normal (7) | 0.035 | 0.891 |
| Adequate _Normal (3) | Adequate _Altered (5) | 0.035 | 0.503 |
| Adequate _Normal (3) | Inadequate _Altered (18) | 0.043 | 0.171 |
| Inadequate _Normal (7) | Inadequate _Altered (18) | 0.508 | 0.167 |
| Inadequate _Normal (7) | Adequate _Altered (18) | 0.968 | 0.012 |
| *Pregestational BMI + GWG + Neurodevelopment* | | | |
| Normal dyads (4) | Altered dyads (5) | 0.05 | 0.855 |

**Note:** Pairwise comparisons of placental DA concentrations between groups defined by pregestational BMI (normal weight, obesity), GWG (adequate, inadequate), and infant neurodevelopmental status (normal vs altered). p-values were calculated using the Kruskal-Wallis test for all comparisons except for the analysis of Normal vs Altered dyads, where a Student’s t-test was applied due to normality assumptions being met. Effect sizes are reported as r (Rosenthal’s r), calculated from the standardized test statistic (Z/√N) for non-parametric comparisons, and derived from the t statistic for the parametric analysis. This metric reflects the magnitude of differences in placental DA concentrations between groups. Effect sizes were interpreted as small (r ≈ 0.1), moderate (r ≈ 0.3), and large (r ≥ 0.5). Given the small sample sizes in several comparisons, effect size estimates should be interpreted cautiously within an exploratory framework.

**Table S5.** Pairwise comparisons of placental dopaminergic protein expression between control and altered dyads, including effect size estimates.

| **Protein** | **p_value** | **Effect size** |
| --- | --- | --- |
| TH | 0.565 | 0.088 |
| COMT | 0.014 | 0.406 |
| VMAT2 | 0.039 | 0.691 |
| OCT3 | 0.714 | 0.657 |
| DAT | 0.002 | 1 |
| DRD2 | 0.3307 | 0.195 |

**Note:** Pairwise comparisons of placental dopaminergic protein expression (TH, COMT, VMAT2, OCT3, DAT, and DRD2) between normal and altered mother–infant dyads. p-values were calculated using either the Mann–Whitney U test or Student’s t-test, depending on data distribution and variance assumptions. Effect sizes are reported as r (Rosenthal’s r), calculated from the standardized test statistic (Z/√N) for non-parametric comparisons, and derived from the t statistic for parametric analyses. Effect size interpretation: small (r ≈ 0.1), moderate (r ≈ 0.3), and large (r ≥ 0.5). Given the limited sample size in several comparisons, results should be considered exploratory.
